# Aptamers Structure Flexibility Is Crucial to EGFR Inhibition Function

**DOI:** 10.3390/ph19081246

**Published:** 2026-08-07

**Authors:** Yahya Sliman, Valeria Ivko, Lika Fab, Fatima Dzarieva, Nadezhda Samoylenkova, Ekaterina Savchenko, Sergey Drozd, Olga Kalennik, Andrey Golovin, Alexey Kopylov, Dmitry Usachev, Galina Pavlova

**Affiliations:** 1 Institution N. N. Burdenko National Medical Research Center of Neurosurgery of the Ministry of Health of the Russian Federation, 125047 Moscow, Russia; valerian.moiseenko@gmail.com (V.I.); dz.fatima@mail.ru (F.D.); samoylenkova.n@gmail.com (N.S.); savhenko61@mail.ru (E.S.); sdrozd@yandex.ru (S.D.); kalennik-olga@mail.ru (O.K.); kopylov.alex@gmail.com (A.K.); dousachev@nsi.ru (D.U.); 2 Moscow Institute of Physics and Technology, National Research University, 141701 Dolgoprudny, Russia; 3 Belozersky Research Institute of Physical Chemical Biology, Lomonosov Moscow State University, 119991 Moscow, Russia; golovin.andrey@gmail.com; 4 Department of Chemistry, Lomonosov Moscow State University, 119991 Moscow, Russia; 5 Institute of Higher Nervous Activity and Neurophysiology, Russian Academy of Sciences, 117485 Moscow, Russia; lika.fab7@gmail.com; 6 Faculty of Bioengineering and Bioinformatics, Lomonosov Moscow State University, GSP-1, Leninskiye Gory, 1-73, 119234 Moscow, Russia; 7 Department of Medical Genetics and Postgenomic Technologies, Sechenov First Moscow State Medical University, 119991 Moscow, Russia

**Keywords:** glioblastoma, aptamer, EGFR, antiproliferative activity, aptamer truncation, apoptosis, aptamer GR20

## Abstract

**Background/Objectives:** Glioblastoma is an aggressive brain tumour with a poor prognosis and limited therapy. The truncated aptamer GR20, derived from aptamer U31, was developed to retain EGFR binding while reducing size. This study evaluated how aptamer truncation, while maintaining its tertiary structure, affects its antiproliferative activity against human glioma cells. **Methods:** Five transplantable human glioblastoma cell cultures (BU73, G22, G23, G01, Sus/fP2) were used. Aptamer binding was assessed by flow cytofluorimetry, intracellular accumulation was tracked by confocal microscopy. Changes in stemness and malignancy gene expression were evaluated by real-time PCR. Protein expression levels were assessed by immunocytochemistry. Proliferative activity was measured using the MTS assay. **Results:** GR20 retained EGFR binding and demonstrated more than two-fold higher binding efficiency to glioblastoma cells than antibodies or the U31 aptamer. Both aptamers accumulated in cells after 2 h of incubation. GR20 significantly reduced the expression of stemness genes (*CD133*, *OCT4*) and malignancy genes (*EGFR*, *PDGFRα*, *TP53*) but showed modest antiproliferative effects (9–11% reduction). U31 exhibited stronger antiproliferative activity, reducing Sus/fP2 viability by 31% and 58%. Molecular dynamics showed that U31 maintains a “multi-loop” conformational state absent in GR20. **Conclusions:** Although the truncated GR20 still binds to the EGFR and is taken up by cells, its antiproliferative activity is reduced compared to U31. The multi-loop conformation present in U31 but missing in GR20 appears to enhance activity, identifying this motif as a promising target for future aptamer optimization.

## 1. Introduction

Despite significant advances in cancer treatment in recent years, oncological diseases remain one of the leading causes of death worldwide. Malignant primary brain tumours in adults are particularly serious, with gliomas representing the most aggressive form. According to the 2021 WHO international classification, supplemented by molecular biomarkers for various tumour types, gliomas are divided into malignancy grades 1 through 4. High-grade gliomas are more malignant and cannot be treated by surgical resection alone [1]. 

A grade 4 glioma with IDH wild-type is called glioblastoma (GBM) and represents the most aggressive brain tumour [1]. The median survival of patients with GBM is approximately 14–17 months [1,2,3]. Despite extensive research conducted over the past several decades, survival rates for GBM have not improved and the prognosis remains poor [4]. The main reasons for the limited effectiveness of treatment (including surgery, radiotherapy and chemotherapy) are as follows: (1) significant tumour heterogeneity, which allows resistant cell subpopulations to survive [5]; (2) GBM-induced immunosuppression, a key tumour defence mechanism [6]; and (3) localization of the tumour in the brain, protected by the blood–brain barrier, which hampers drug delivery [6]. There is therefore a need for novel therapeutic approaches that inhibit glioblastoma growth, reduce the capacity of GBM cells for migration and invasion, and increase their sensitivity to therapy.

One promising glioma marker is the epidermal growth factor receptor (EGFR), a transmembrane protein constituting a receptor tyrosine kinase [7]. Binding of wild-type EGFR to epidermal growth factor (EGF) leads to autophosphorylation of the intracellular domain of the EGFR and triggers a phosphokinase cascade. Phosphokinase activates several transcription factors and induces expression of pathological genes [8]. Amplification of the EGFR gene is a key driver and prognostic factor, occurring in approximately 57% of primary glioblastoma cases in adults. Tumours with EGFR amplification have been shown to be more aggressive but may be vulnerable to targeted therapy directed at this receptor [9,10]. Other forms of genetic alteration in the EGFR have also been reported, including gene rearrangements, point mutations, and deletions. One notable example is the deletion of exons 2–7, which produces the truncated mutant variant EGFRvIII [11]. This variant lacks 267 amino acids within the extracellular domain and remains constitutively active in the absence of EGF binding, resulting in uncontrolled cellular proliferation [8].

The major biological importance of EGFR and its mutant variants has driven the development of therapeutic and diagnostic molecules capable of specifically binding to, inhibiting, or distinguishing between different EGFR forms. For example, several EGFR-specific aptamers have been successfully developed for the recognition of cells expressing this receptor. Aptamers are unique synthetic single-stranded DNA or RNA molecules capable of forming secondary and tertiary structures [12]. They are distinguished by their small size, stability and ease of synthesis. Because they are produced by chemical synthesis, their composition and purity can be precisely controlled, and they are minimally immunogenic [13]. These characteristics make them potential candidates for binding to target molecules such as peptides, proteins, metal ions, bacteria, viruses and other targets [14]. The field of application for aptamers is expanding due to their enormous potential: anti-tumour activity, temporal stability in the bloodstream, biocompatibility, multimodal diagnostic functions and the possibility of delivering an effective payload [14,15].

To date, more than 30 aptamers targeting various EGFR proteins have been described [16,17]. A number of these aptamers are able to discriminate between wild-type EGFR (EGFRwt) and the mutant variant EGFRvIII. For example, aptamer CL4 binds to both EGFRwt and EGFRvIII [18,19], whereas aptamer ME07 shows preferential binding to EGFRwt over EGFRvIII [20]. In contrast, aptamer Gol1 demonstrates the highest affinity for EGFRvIII [21,22]. These aptamers can also serve as carriers for a range of functional payloads. They may be used to inhibit EGFR-dependent signalling pathways or to deliver therapeutic agents selectively to EGFR-positive cells [23]. More recently, considerable attention has been directed towards the application of EGFR-specific aptamers for targeted delivery of therapeutic compounds directly to tumour cells [24], as well as for the visualization of EGFR-expressing cells both in vitro and in vivo [25].

Taken together, these findings support the potential use of aptamers as promising tools for the imaging and/or treatment of glioblastoma and may ultimately contribute to improved therapeutic outcomes for patients with malignant brain tumours.

The development of new aptamers is often carried out by the SELEX method, selection from a library of oligonucleotides containing a randomized region [26]. Another approach involves modifications and truncations of already-known aptamers, controlled to preserve the tertiary structure required for interaction with the target protein. In order to obtain a truncated variant, the 5′- and 3′-terminal nucleotide sequences 1–10 and 57–76 were deleted from the previously described aptamer U31 [27]. These terminal sequences are not expected to be involved in the formation of the predicted functional three-dimensional structure of the aptamer. Removal of the terminal regions reduces the length of the aptamer from 76 to 47 nucleotides, which simplifies chemical synthesis and can potentially positively affect pharmacokinetic properties. Nucleotide 16G was replaced by 16C, which increased the duplex from 5 to 8 nucleotide pairs and led to its stabilization without disrupting the central loop, which may be involved in interaction with EGFR [23]. Using this method, the truncated DNA aptamer GR20 was obtained and its ability to bind the extracellular domain of EGFR was demonstrated [23,28]. It has been suggested that this modification of GR20 increases the conformational stability of the aptamer, which leads to slower association, but at the same time, delayed dissociation of the aptamer-EGFR complex, which maintains approximately the same constant value (24 ± 6 nM for U31 versus 26 ± 5 nM for GR20) [28]. At the same time, according to the results of affinity analysis using the fluorescence polarization method, the truncated aptamer GR20 binds 4.5 times better than the original aptamer U31 (aK_D_ = 3.5 ± 0.1 nM versus 15.9 ± 9.2 nM) [28]. Shortening the aptamer may improve penetration through barriers (e.g., the BBB), facilitate faster internalization into the target cell and, as a result, accelerate the cellular response. The potential of truncated aptamers was demonstrated in the work of Dzarieva et al.: the authors demonstrated that the aptamer Gol1 had a suppressive effect on the expression of genes associated with cell proliferation and cell survival, while the full-length aptamer U2 had no significant effect on cells under the same conditions [29]. Although it has previously been shown that truncation the U31 aptamer to GR20 maintains its affinity for the EGFR, the effect of this modification on biological activity in glioma cells has not been studied. This study aims to fill this gap. We hypothesize that truncating the aptamer, while maintaining its tertiary structure, can affect its cellular accumulation and antiproliferative effect. In this study, for the first time, we systematically compare the antiproliferative activity of the full-length U31 aptamer and its truncated variant, GR20, on a panel of human glioblastoma cultures. We demonstrate the impact of these aptamers on changes in the expression of stemness and malignancy genes, as well as protein expression. Furthermore, we hypothesize factors (structural stability and flexibility) that may determine differences in the biological efficacy of the aptamers, as we have shown for the first time that truncation of the aptamer, while maintaining its affinity for the EGFR and the ability to internalize, can lead to a decrease in antiproliferative activity. These results open new possibilities for the rational design of therapeutic aptamers.

## 2. Results

### 2.1. PCR Analysis of EGFR Expression in the Cell Cultures Studied

**Figure 1:**
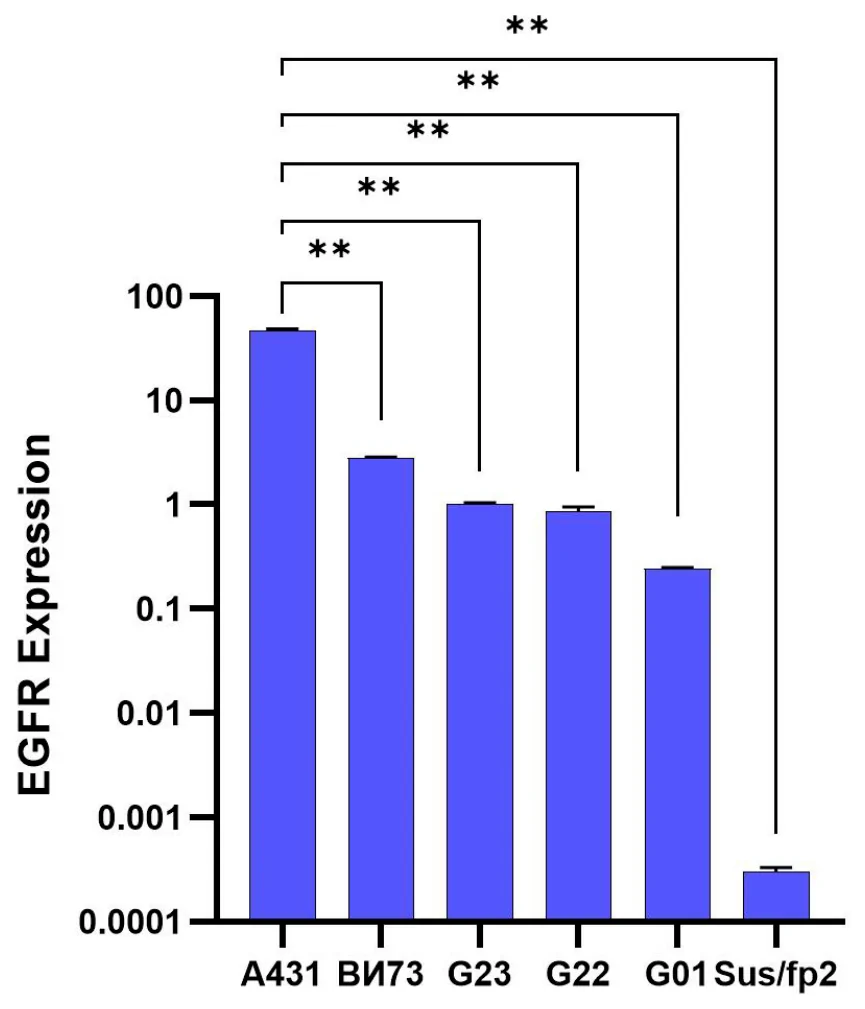
The level of EGFR expression in A431 adenocarcinoma cells and in human glioblastoma culture cells BU73, G23, G22, G01 and Sus/fP2. Data are presented as the mean ± SD; *n* = 3 per group. Statistically significant differences between groups are indicated by asterisks (multiple unpaired *t*-tests, ** = *p* < 0.01).

Human glioblastoma cell cultures obtained from the Biobank of the N.N. Burdenko National Medical Research Centre for Neurosurgery, BU73, G23, G22, G01, and Sus/fP2 [30], were selected for this study. EGFR expression levels in these cultures were evaluated using quantitative PCR. The A431 cell line, characterized by high EGFR expression, served as a positive control (Figure 1).

EGFR expression in the BU73, G23, G22, and G01 glioblastoma cultures was found to be 10–100 times lower than that observed in A431 cells. The Sus/fP2 culture exhibited the lowest EGFR expression level, approximately six orders of magnitude lower than that of A431 cells.

### 2.2. Evaluation of the Binding Efficiency of Aptamers U31 and GR20 to A431 Cells and Human Glioblastoma Cultures with Different Levels of EGFR Expression

**Figure 2:**
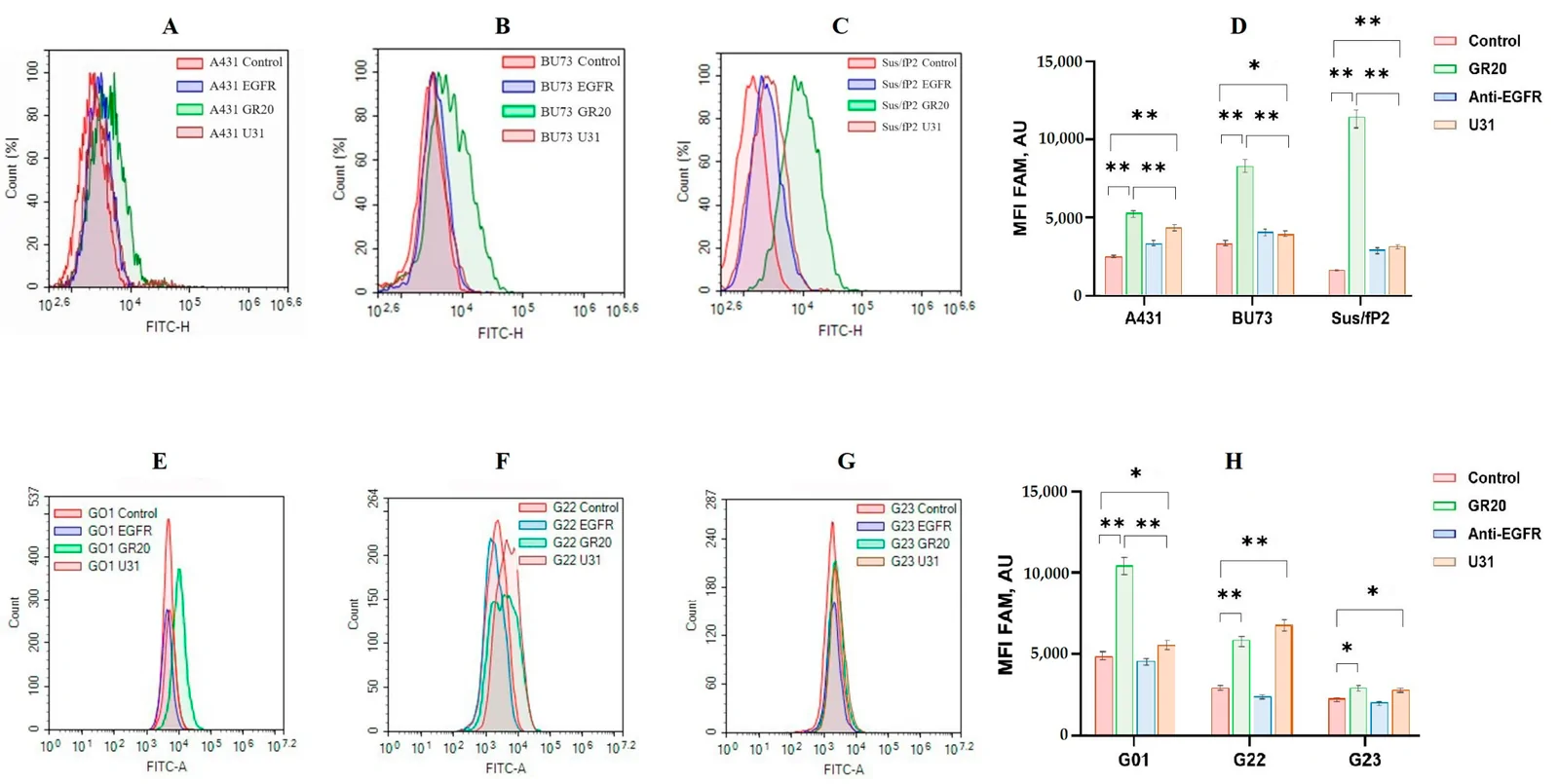
Evaluation of the specificity of aptamers GR20 and U31 for the EGFR in the (**A**) A431 epidermoid carcinoma cell line and in passaged human glioblastoma cell cultures (**B**) BU73, (**C**) Sus/fP2, (**E**) G01, (**F**) G22, and (**G**) G23. Panels (**D**) and (**H**) show the mean fluorescence intensity (MFI) for untreated cells and cells incubated with probes: (**D**) for A431, BU73, Sus/fP2; (**H**) for G01, G22, G23. Data are presented as the mean ± SD; *n* = 3 per group. Statistically significant differences between groups are indicated by asterisks (multiple unpaired *t*-tests, * = *p* < 0.05, ** = *p* < 0.01).

Assessment of the binding efficiency of aptamers U31 and GR20 to their EGFR target was performed on the human epidermoid carcinoma tumour cell line A431 with high *EGFR* expression [31] and on passaged human glioblastoma cultures with different *EGFR* expression levels derived from patient tumour tissue (BU73, Sus/fP2, G01, G22 and G23) by flow cytometry at room temperature for 30 min (Figure 2).

A431 cells and glioblastoma cell cultures were incubated with fluorescently labelled aptamers carrying a FAM fluorophore at the 5′ end (green fluorescence). The obtained data demonstrated that aptamer GR20 exhibited higher binding efficiency than U31 toward the A431 cell line as well as the passaged human glioblastoma cultures BU73, G01, and Sus/fP2. This was evidenced by greater shifts in fluorescence histograms (Figure 2A–H) and higher mean fluorescence intensity (MFI) values. In BU73 and G01 cells, the MFI values for aptamer GR20 were approximately 1.9-fold higher than those observed for aptamer U31. In contrast, both aptamers demonstrated nearly identical binding efficiency in the passaged human glioblastoma cultures G22 and G23 (Figure 2F–H).

### 2.3. Investigation of Effect of Aptamers U31 and GR20 on Gene Expression in Human Glioblastoma Cultures Sus/fP2 and G01

**Figure 3:**
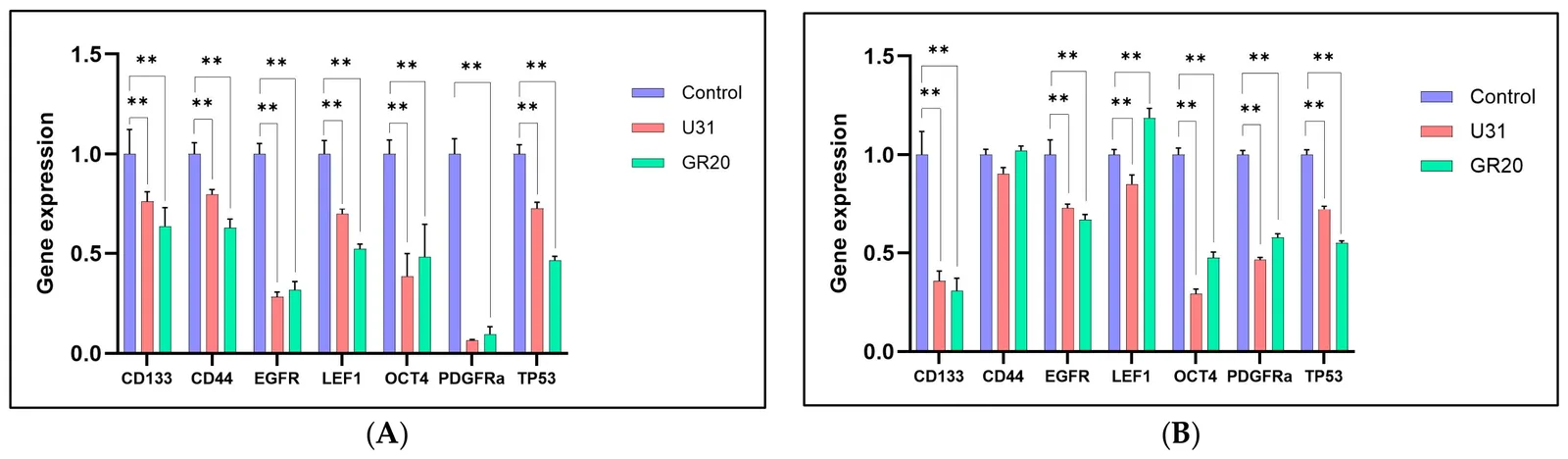
Effect of aptamers U31 and GR20 on gene expression changes in human glioblastoma culture cells (**A**) Sus/fP2 and (**B**) G01. Data are presented as mean ± SD; *n* = 3 per group. Statistically significant differences between groups are indicated by asterisks (multiple unpaired *t*-tests, ** = *p* < 0.01).

The effect of aptamers U31 and GR20 on the expression of stemness and malignancy genes in human glioblastoma culture cells was assessed by qPCR (Figure 3A,B).

Treatment of human glioblastoma culture Sus/fP2 cells with the aptamer GR20 resulted in decreased expression of stemness-associated genes, including CD133, CD44, and OCT4, as well as malignancy-related genes such as EGFR, LEF1, and TP53, compared with the control sample. The most pronounced suppression was observed for PDGFRα expression (Figure 3A). In the human glioblastoma culture G01, exposure to aptamer GR20 also led to reduced expression of the stemness genes CD133 and OCT4, together with decreased expression of the malignancy-associated genes EGFR, PDGFRα, and TP53.

Overall, a reduction in expression of several stemness- and malignancy-related genes was found following treatment with the aptamers. However, no statistically significant differences between these aptamers were observed when comparing their effect on gene expression in human glioblastoma culture cells.

### 2.4. Investigation of Efficacy of Aptamers U31 and GR20 on Human Glioblastoma Cells

**Figure 4:**
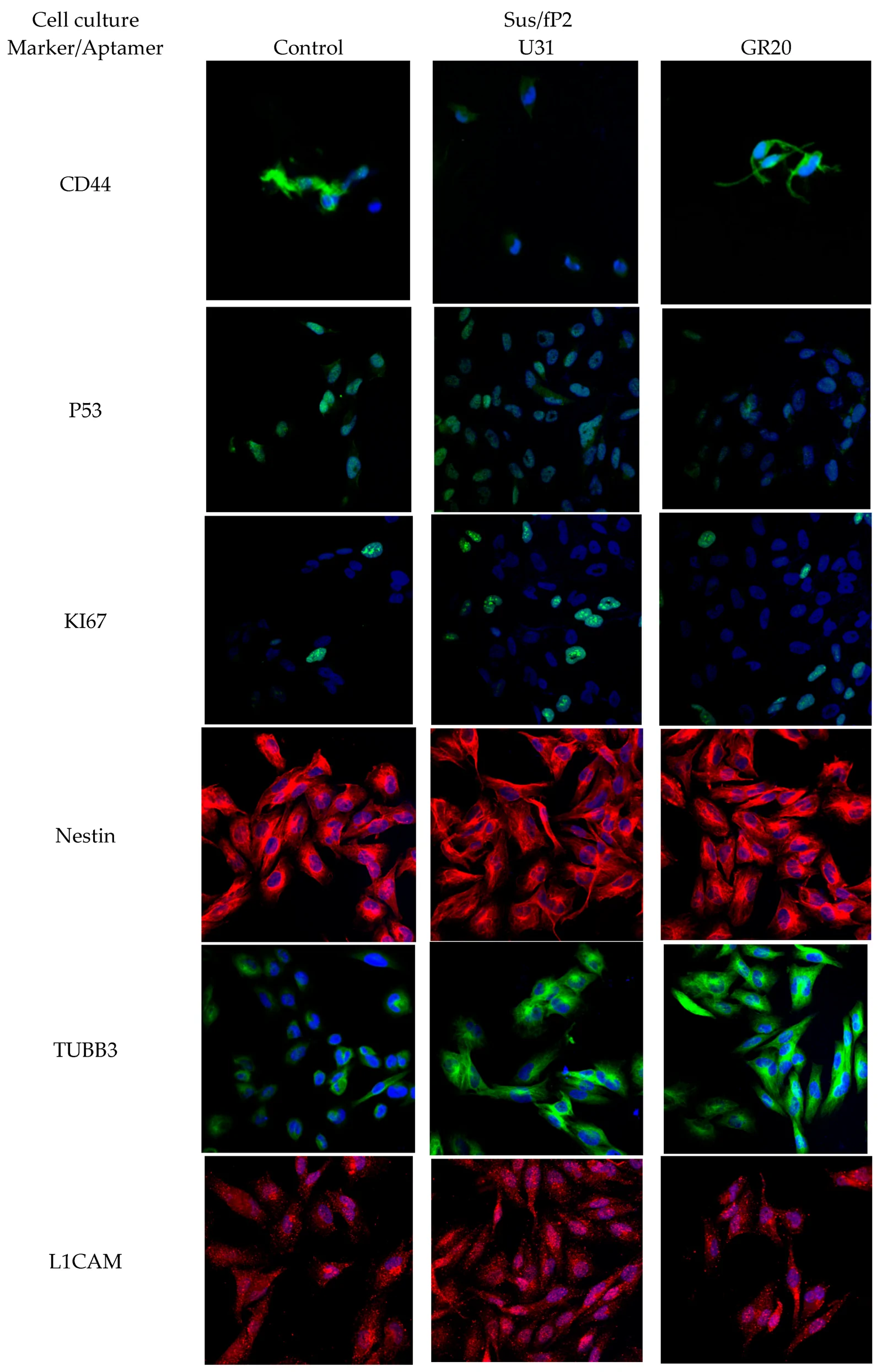
Immunocytochemical analysis of the effects of aptamers GR20 and U31 on the Sus/fP2 glioblastoma cell culture. Nuclei stained with bisbenzimide (Hoechst 33342, Sigma, Burlington, MA, USA) are shown in blue.

The obtained effect of aptamers on RNA levels in glioblastoma cells required further analysis via immunocytochemical methods. The effect of aptamers U31 and GR20 on human glioblastoma cell cultures Sus/fP2 and G01 was assessed by immunocytochemistry (ICC). Based on immunocytochemical staining of human glioblastoma culture Sus/fP2 cells, it was found that prior incubation of cells with aptamers leads to reduced representation of CD44; the ICC staining intensity was substantially lower in the case of U31 (Figure 4). Similar results were obtained upon staining Sus/fP2 cells with antibodies to P53 and Ki67; however, the ICC staining intensity was substantially lower in the case of GR20. The effects of U31 and GR20 on the representation of markers Nestin, βIII-tubulin and L1CAM in Sus/fP2 culture cells were also analyzed. Both aptamers lead to increased representation of βIII-tubulin in glioblastoma cells (Figure 4), suggesting that tumour cells tend to transition to a neuroblast stage following treatment with the aptamers studied.

The observed effects of the aptamers on RNA expression levels in glioblastoma cells required further investigation using immunocytochemical methods. The influence of aptamers U31 and GR20 on the human glioblastoma cell cultures Sus/fP2 and G01 was therefore evaluated by immunocytochemistry (ICC). Immunocytochemical staining of Sus/fP2 cells demonstrated that pre-incubation with the aptamers reduced CD44 representation, with a markedly lower staining intensity observed following treatment with U31 (Figure 4). Similar results were obtained for staining with antibodies against P53 and Ki67. In addition, the effects of U31 and GR20 on the expression of the markers Nestin, βIII-tubulin, and L1CAM in Sus/fP2 cells were analyzed. Both aptamers increased the representation of βIII-tubulin in glioblastoma cells (Figure 4), suggesting a tendency of tumour cells to transition toward a neuroblast-like state following aptamer treatment.

**Figure 5:**
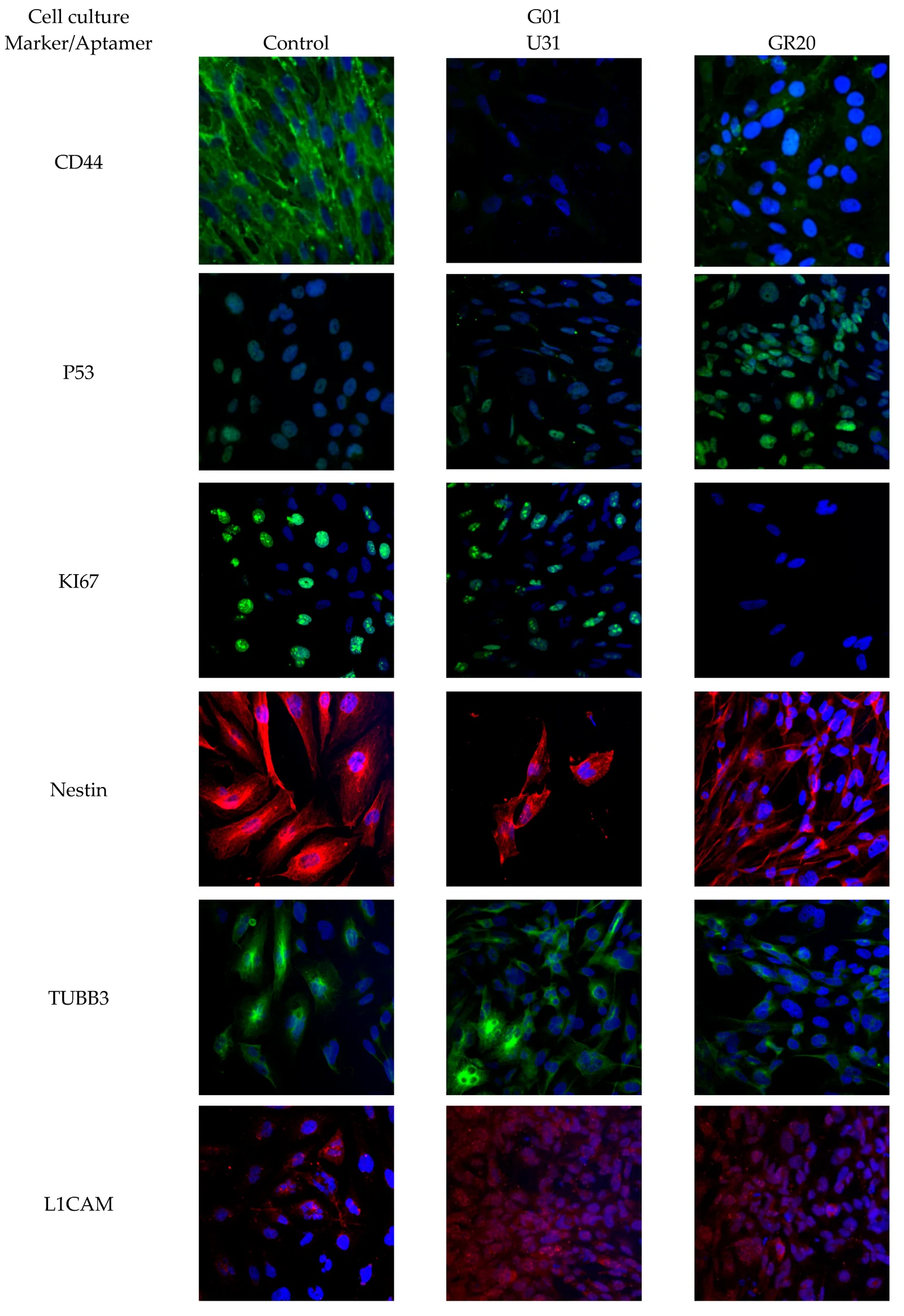
Immunocytochemical analysis of the effects of aptamers GR20 and U31 on the G01 glioblastoma cell culture. Nuclei stained with bisbenzimide (Hoechst 33342, Sigma, Burlington, MA, USA) are shown in blue.

In human glioblastoma G01 cells, similarly to the Sus/fP2 culture, aptamer treatment resulted in a marked reduction in CD44 representation, as evidenced by decreased fluorescence staining intensity. Following treatment with U31, fluorescence associated with antibody labelling became nearly undetectable. In addition, treatment of G01 cells with the aptamers reduced the representation of Ki67, βIII-tubulin, and Nestin in tumour cells, with the most pronounced effects observed for aptamer GR20. Altered localization of the L1CAM protein was also detected in G01 cells and was characterized by its location in the nucleus (Figure 5). Changes in the cellular localization of other proteins were minor.

### 2.5. Assessment of U31 and GR20 Accumulation in Human Glioblastoma Cells

**Figure 6:**
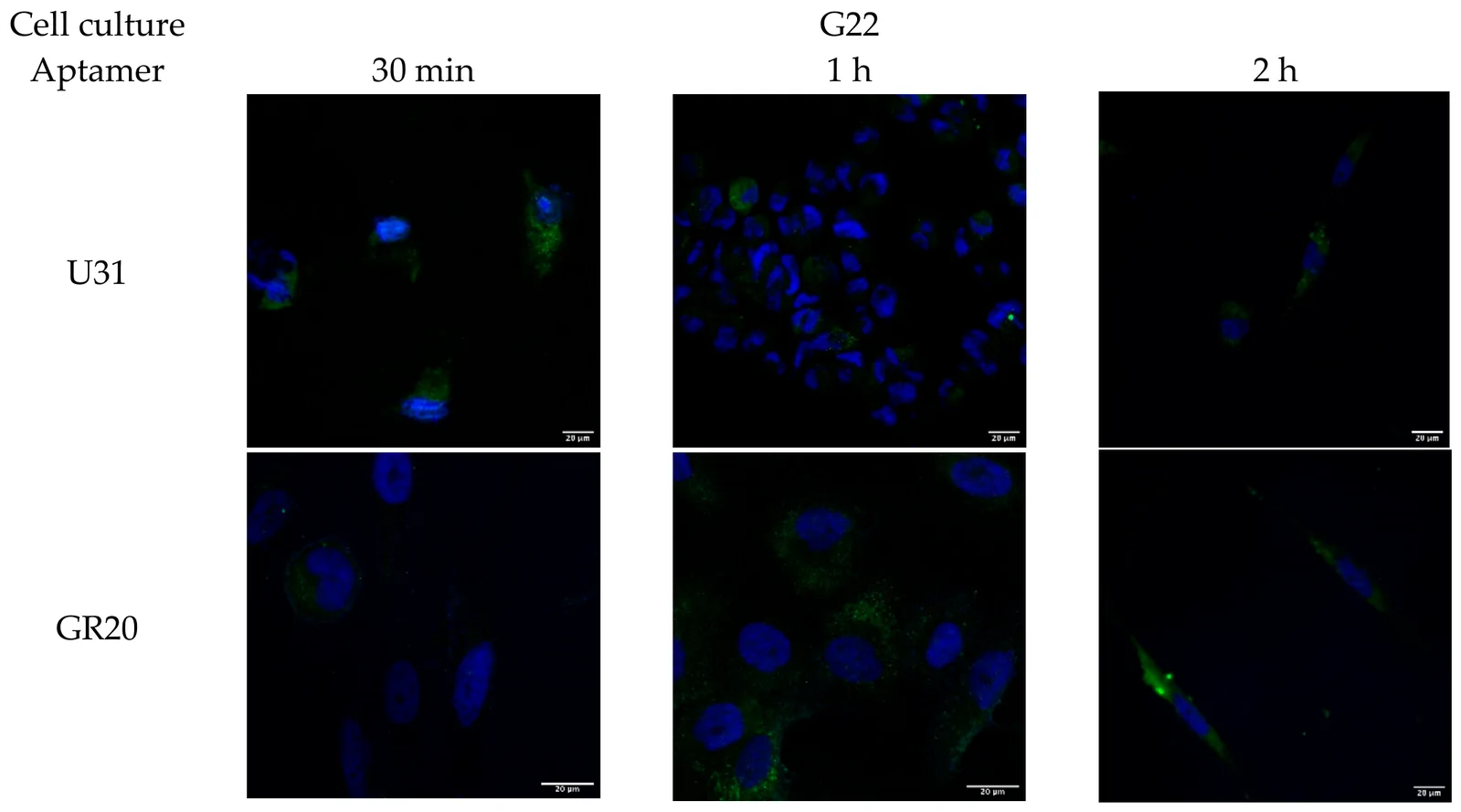
The accumulation and intracellular distribution of aptamers U31 and GR20 (1 µM) in primary G22 glioblastoma cells after 30 min, 1 h, and 2 h of incubation, as assessed by confocal microscopy. FAM-labelled aptamers are shown in green, and nuclei stained with bisbenzimide (Hoechst 33342, Sigma, Burlington, MA, USA) are shown in blue.

To evaluate aptamer penetration and accumulation in tumour cells, time intervals of 30 min, 1 h, and 2 h were used. The study was performed using two human glioblastoma cell cultures with high EGFR expression, G01 and G22. Granular cytoplasmic accumulation of aptamer U31 in G22 cells was observed at earlier time points, namely after 30 and 60 min of incubation, whereas accumulation of aptamer GR20 was detected only after 60 min. After 2 h of incubation, both aptamers were present in the cells (Figure 6).

**Figure 7:**
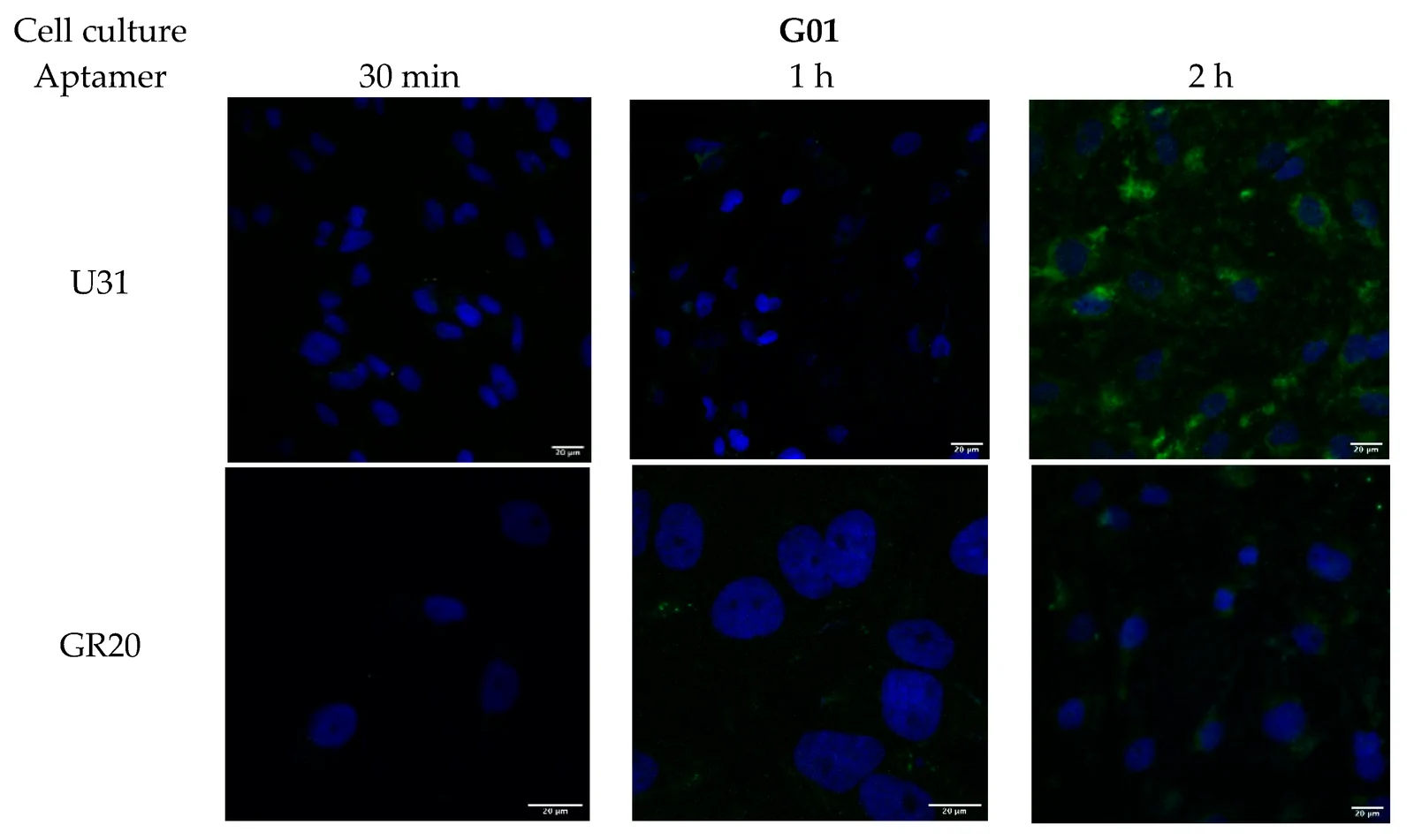
Accumulation of aptamers U31 and GR20 (1 µM) in primary glioblastoma culture G01 cells after 30 min, 1 h and 2 h. Confocal microscopy. FAM-labelled aptamers are shown in green, and nuclei are counterstained with bisbenzimide (Hoechst 33342; Sigma) in blue.

A similar pattern was observed in G01 glioblastoma cells. In the cytoplasm of G01 culture cells, weak accumulation of aptamer U31 was detected after 30 min of incubation, whereas the accumulation of aptamer GR20 was observed only after 60 min. After 2 h of incubation, both aptamers were present in the cells; however, the accumulation intensity was greater for aptamer GR20 (Figure 7). These findings indicate that both aptamers can penetrate the cell membrane, although their rates of internalization differ, with U31 entering the cells more rapidly.

### 2.6. An Assessment of the Effect of Aptamers U31 and GR20 on the Proliferative Activity of Passaged Human Glioblastoma Culture Cells

**Figure 8:**
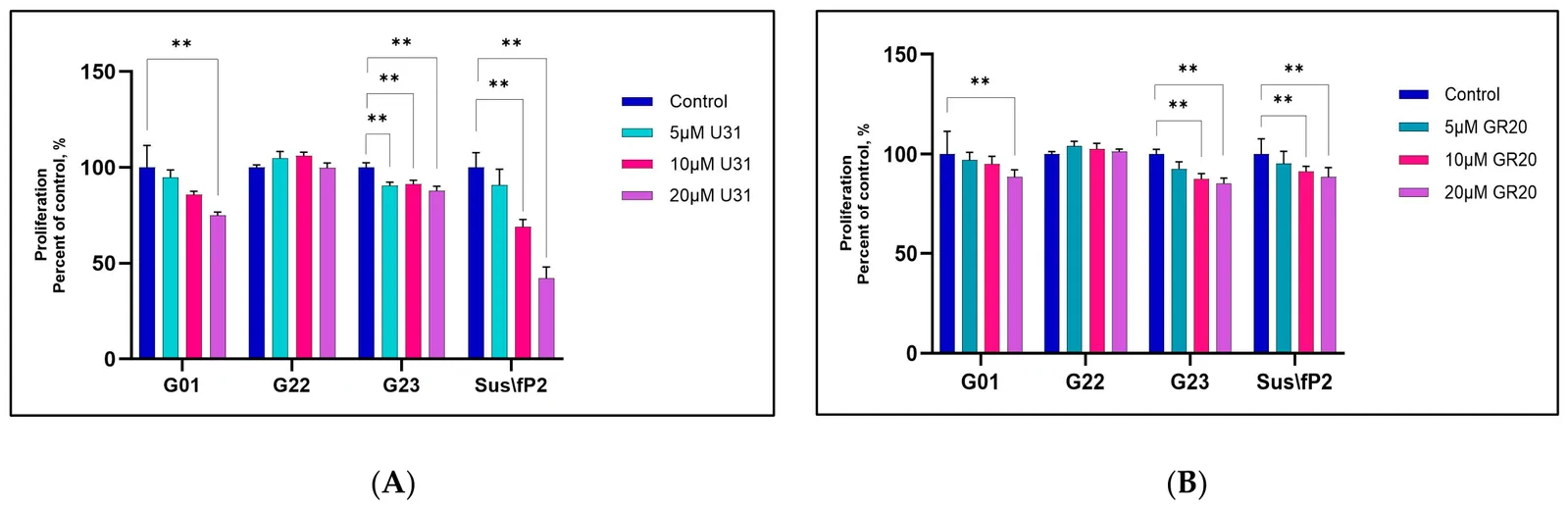
Proliferative activity of glioblastoma cell cultures G01, Sus/fP2, G22, and G23 following treatment with aptamers U31 (**A**) and GR20 (**B**) at concentrations of 5 µM, 10 µM, and 20 µM. Data are presented as mean ± SD (n = 3 per group). Statistically significant differences between groups are indicated by asterisks (multiple unpaired *t*-tests; ** = *p* < 0.01).

Cell proliferative activity was assessed using the MTS assay. The analysis was conducted on human glioblastoma cell cultures G01, G22, G23, and Sus/fP2. Aptamers were added at three different concentrations: 5 µM, 10 µM, and 20 µM. The results for aptamers U31 and GR20 are presented in Figure 8.

Treatment with the aptamer U31 at all tested concentrations produced a statistically significant (*p* ≤ 0.01) decrease in the metabolic activity of G23 cells compared with the control, by 9.5%, 9%, and 12% at 5, 10, and 20 µM, respectively (Figure 8A). In Sus/fP2 cell culture, U31 showed a more pronounced antiproliferative effect, with cell proliferation reduced by 31% and 58% at 10 µM and 20 µM, respectively (*p* ≤ 0.01). In G01 cells, a significant reduction in metabolic activity (25% compared with control, *p* ≤ 0.01) was observed only at the highest concentration (20 µM), while no significant changes were detected at lower concentrations. No statistically significant effect of U31 was observed in G22 cells.

Treatment of cells with the aptamer GR20 (Figure 8B) led to a significant reduction in the proliferation of G01 cells (11.5%, *p* ≤ 0.01), observed only at 20 µM, with no effect at lower concentrations. In Sus/fP2 glioblastoma culture cells, a reduction in metabolic activity of 9% and 11% compared with the control was observed after incubation with aptamer at concentrations of 10 and 20 µM, respectively. The most pronounced antiproliferative effect was observed in G23 cells, where GR20 induced a dose-dependent reduction in metabolic activity of 7%, 12%, and 14.5% across increasing concentrations.

In contrast, G22 glioblastoma cells were insensitive to treatment with aptamers U31 and GR20, as no statistically significant differences relative to the control group were observed (Figure 8).

### 2.7. Assessment of Secondary Structure of U31

**Figure 9:**
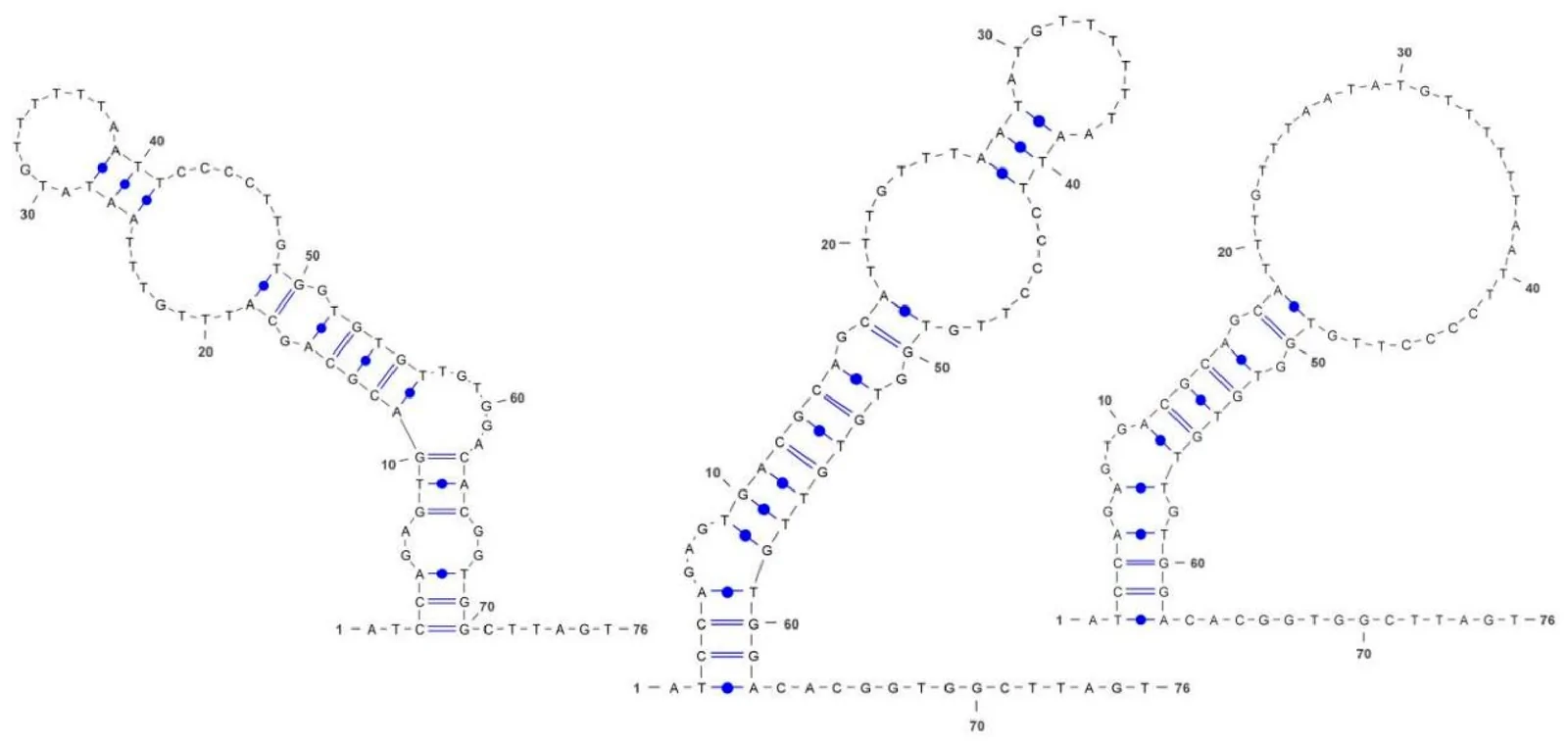
Predicted secondary structure variants of DNA aptamer U31.

Based on predictions obtained using ViennaRNA with DNA-specific parameters [32], three secondary structure variants of U31 with similar free energies were identified (Figure 9). From a deletion analysis perspective, these variants are largely equivalent, as they all contain conserved 3′- and 5′-terminal regions corresponding to the primer binding sites used during the SELEX procedure.

**Figure 10:**
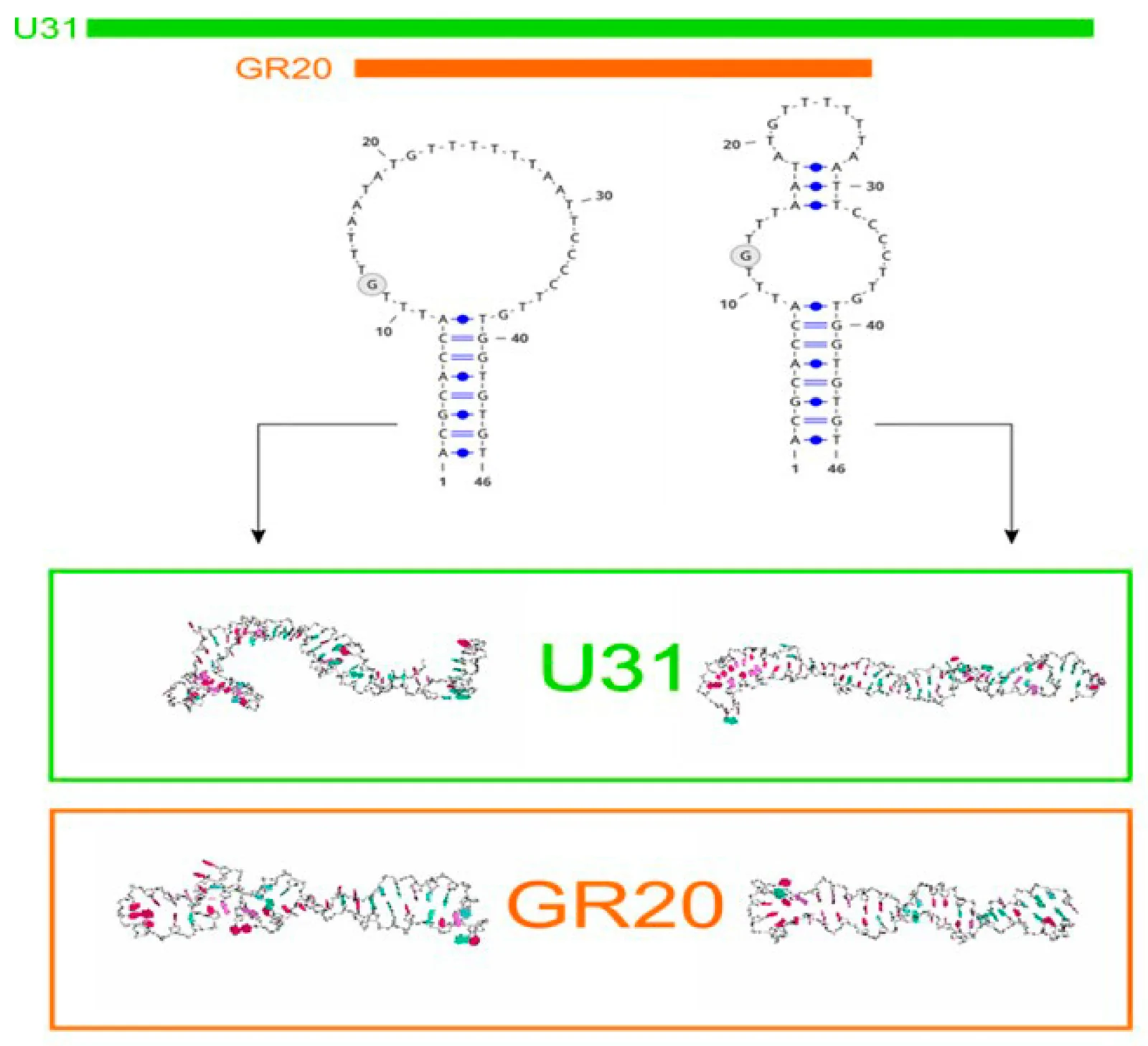
Schematic representation of computational analysis of structural models of aptamers U31 and GR20.

The proposed secondary structures of the aptamer GR20 contain the same secondary structure pattern which is very close to the formation energies of the original aptamer U31 (Figure 10). However, assessment of the proposed secondary structures does not allow for an unambiguous selection of a structural model for further study. Therefore, a comparative study of the structural models of U31 and GR20 was performed based on two variants of the secondary structure of the central loop, unstructured and structured (Figure 10). Four initial models were built using DNA modelling parameters [33]. 

Scanning of the conformational space of models was performed using the metadynamics method [34]; eRMSD [35] was used as the collective variable for residues belonging to the central loop. eRMSD is a modification of the well-known RMSD metric adapted for the analysis of nucleic acids. After completion of potential addition, a long molecular dynamics simulation with constant potential was performed. The resulting conformational diversity was studied using cluster analysis based on RMSD values for all DNA atoms. For aptamers U31 and GR20, the first 3–4 clusters contained the main number of conformations observed during the simulation (Table 1).

Visual analysis of the aptamer conformations revealed two predominant structural forms: an “overall helical” conformation and a “multi-loop” conformation. GR20 was represented predominantly by the overall helical form, whereas U31, when modelled with an unstructured central loop, showed two of the three largest clusters adopting the multi-loop conformation. Although only limited structural data are currently available, comparison of the simulation results with the biological activity of different U31 variants suggests that the structured large loop represents the key functional element of the DNA secondary structure. Cluster analysis further demonstrated that this structural variant exhibits greater conformational diversity, while the “hairpin” variant is represented by only a single dominant secondary structure. Truncation of the aptamer U31 to generate GR20 resulted in a marked reduction in the representation of the multi-loop (junction) structural element, which is likely the primary reason for the lower biological activity of GR20 compared with U31.

## 3. Discussion

Investigation of the antiproliferative activity of aptamers represents an important step in the development of aptamer-based anti-tumour agents. Aptamers have advantages in toxicological profile and ADME properties (absorption, distribution, metabolism, excretion) compared with conventional small-molecule drugs. However, the development of antiproliferative aptamers is associated with challenges related to their conformational mobility. The aim of this study was to find out how truncation of the anti-EGFR aptamer U31, while maintaining its predicted tertiary structure, affects antiproliferative activity against human glioblastoma cells. The results obtained demonstrate that the truncated version of GR20, despite maintaining and even improving its affinity for the EGFR, as well as its ability to internalize, has significantly reduced antiproliferative activity compared to the full-size U31. This observation raises an important question about which properties of the aptamer, in addition to binding affinity, determine its functional activity.

A slower dissociation rate of the aptamer GR20-EGFR (recombinant protein) complex was previously demonstrated, with the dissociation constant (aK_D_) value retained (24 ± 6 nM for U31 and 26 ± 5 nM for GR20, as demonstrated by the BLI method). At the same time, according to the results obtained by using the fluorescence polarization method, aK_D_ for GR20 is 4.5 times lower than that for the full-length U31 aptamer [28]. These data indicate a comparable or higher affinity of the truncated variant. Notably, according to flow cytometry data, the aptamer GR20 demonstrated more than twofold greater binding efficiency to glioblastoma cell cultures compared with both antibodies and the aptamer U31 (Figure 2). At first glance, these data, together with the increased affinity of GR20, contradict its weaker antiproliferative effect. However, such a discrepancy between affinity and functional activity is not unique to aptamers and has been described for a number of other molecules, including monoclonal antibodies [36,37]. This indicates that the key importance is not just the fact of binding, but the ability of the molecule to influence the conformational state and/or dimerization of the receptor, which, in turn, determines the nature of downstream signalling. It can be assumed that U31, due to the presence of a multi-loop conformation, stabilizes the EGFR in an inactive state or prevents its ligand-dependent dimerization, whereas GR20, binding to the receptor, does not induce the necessary structural rearrangements. This assumption is consistent with the data that different epitopes on EGFR can have different effects on its activation [38].

The results of molecular dynamic modelling presented in this paper provide a structural basis for explaining the functional differences. It is shown that U31 is capable of adopting a “multi-loop” (multi-loop/junction) conformation, which is represented in the second most populated cluster, while GR20 has practically no such conformation: it exists mainly in a “general spiral” form. The loss of the ability to form a multi-loop structure upon truncation is probably the main reason for the decrease in antiproliferative activity (Figure 8), despite the preserved affinity. This observation is consistent with previously published data that conformational plasticity is critically important for the functional activity of nucleic acids [39] and identifies a specific structural motif that can serve as a target for further optimization of therapeutic aptamers. A similar dependence of biological activity on certain conformational states is described, in particular for the thrombin aptamer forming the G-quadruplex [40].

Special attention should be paid to the observed binding of both antibodies and aptamers to Sus/fP2 glioblastoma cells despite their low EGFR mRNA expression levels (Figure 1 and Figure 2). It may be associated with a significant contribution of membrane-mediated interactions. Similar phenomena have been described previously [41,42,43]. It is worth noting that the antiproliferative effect of U31 was also most pronounced in Sus/fP2 culture (a reduction in metabolic activity of 31% and 58% at concentrations of 10 µM and 20 µM, respectively) (Figure 8), which, according to PCR data, has the lowest level of EGFR expression among the studied cell cultures (Figure 3A). This unexpected result may have several explanations. First, cells with low EGFR levels may be more dependent on this receptor for survival and proliferation, making them more sensitive to EGFR-directed therapy—a phenomenon described in the literature as “Oncogene Addiction” [44]. Secondly, differences in the mutational status of downstream EGFR effectors (PTEN, PI3KCA, KRAS) may determine how critical EGFR signalling is for a particular cell culture. Cultures G01, G22, and G23 may carry activating mutations in the underlying pathways, which makes them less sensitive to EGFR blocking, regardless of its expression level.

Comparison of qPCR and immunohistochemical analysis results of gene expression upon treatment of human glioblastoma culture G01 with aptamers U31 and GR20 reveals an evident result: protein expression changes more slowly than RNA expression. Our data on changes in the expression of stem cell genes (CD133, OCT4, CD44) (Figure 3) and genes associated with malignant potential (EGFR, PDGFRa, TP53) (Figure 3) under the influence of both aptamers deserve a separate discussion. A decrease in the expression of CD133, a well-known marker of glioblastoma stem cells, is of particular interest, since this cell subpopulation is considered responsible for tumour recurrence and resistance to therapy [45]. The fact that both aptamers suppress CD133 expression, but only U31 shows a significant antiproliferative effect, indicates that a decrease in stemness does not always directly correlate with a decrease in proliferation. It can be assumed that GR20, by suppressing stem genes, promotes the “differentiation” of tumour cells into a less aggressive state, which is confirmed by our data on the increased expression of βIII-tubulin, a marker of neuronal differentiation (Figure 4). However, in order to significantly reduce proliferation, effective inhibition of EGFR-dependent signalling pathways is also required, which is achieved only by full-length U31.

The observed change in the intracellular localization of Nestin and L1CAM proteins after treatment with aptamers U31 and GR20 is also noteworthy (Figure 4 and Figure 5). Nestin, a protein of intermediate filaments, is normally localized in the cytoplasm; its appearance in the nucleus may indicate a violation of cytoskeletal assembly and/or a stress response of the cell [46]. Nuclear localization of L1CAM, which is a transmembrane protein, may indicate a violation of intracellular transport or activation of stress-related signalling pathways. Furthermore, immunocytochemical analysis revealed a marked reduction in Ki67 expression following GR20 treatment (Figure 4 and Figure 5). The expression of Ki67 protein is linked to active phases of the cell cycle, and a decrease in Ki67 levels indicates a decrease in cell proliferative activity. Together, these changes confirm that aptamers induce a complex cellular response that goes beyond simple EGFR inhibition. A detailed study of the mechanisms underlying changes in the localization of these proteins is the subject of further research. Analysis of the accumulation of fluorescently labelled aptamers U31 and GR20 demonstrated distinct accumulation patterns between the two aptamers. These differences appeared to depend both on the specific glioblastoma cell culture (differences in expression levels the target, differences in endocytic activity) and on the aptamer itself (differences in structural stability and affinity). It should be noted that the differences in the dynamics of internalization between U31 and GR20 (U31 accumulates faster in cells) can also contribute to the difference in their antiproliferative activity. Nevertheless, increased intracellular accumulation of both aptamers was observed after 2 h of incubation. Based on these findings, we hypothesize that the optimal time point for analyzing the effects of aptamers U31 and GR20 at a concentration of 1 µM on human glioblastoma cells is beyond 2 h of incubation. The mechanism of aptamer internalization remains poorly understood. It is assumed that it can be mediated by receptor-dependent endocytosis, macropinocytosis, or passive diffusion [47]. Different rates of internalization can determine the exposure time of the aptamer to intracellular targets and/or the effectiveness of its degradation in lysosomes, which, in turn, can modulate the biological response. Slower internalization of GR20 may result in it remaining on the cell surface longer, but being less effective in blocking EGFR signalling because it does not induce the necessary conformational changes in the receptor. Further experiments are required to study the internalization mechanisms in detail (e.g., endocytosis inhibitors and at a reduced temperature (4 °C)).

In this study we did not use reference drugs (e.g., cetuximab or erlotinib), since our goal was not to comparatively evaluate the effectiveness of aptamers with existing drugs; we aimed to directly compare the functional activity of the full-length aptamer U31 and its truncated version GR20, attempting to identify the structural determinants influencing the antiproliferative effect. However, such a comparative study with reference drugs will be a next logical step in our work.

The translation of modified and unmodified aptamers into clinical practice opens new possibilities for targeted action on tumour cells while minimizing systemic toxicity. The results obtained in this study dual significance for future studies of aptamers and patient-derived cell cultures aimed at the development and pursuing translation of aptatheranostics in biomedicine. Since the truncated aptamer GR20 exhibits enhanced binding to glioblastoma cells and cellular accumulation. This makes GR20 a promising vector for targeted delivery of therapeutic agents to EGFR-expressing tumour cells. On the other hand, the loss of antiproliferative activity of GR20 demonstrates that simple truncation while maintaining affinity is not sufficient to obtain a therapeutically active molecule. The identified structural motif (the “multi-loop” conformation) in the U31 aptamer may serve as a basis for the rational design of aptamers with improved functional activity. The ability of aptamers U31and GR20 to affect the expression of stemness and malignancy genes opens up opportunities for their use as components of combination therapy.

Study limitations: First, we used only two aptamers—full-length U31 and its truncated version GR20. This does not allow us to draw conclusions about the influence of other potential modifications or aptamers on the antiproliferative properties of glioma cells. Second, the panel of glioblastoma cell cultures studied is limited to five lines, which may not reflect the full diversity of glioblastomas. Third, the lack of direct comparison with clinically used EGFR inhibitors (e.g., cetuximab) under the same conditions does not allow us to quantitatively evaluate the advantage of aptamers over existing drugs. Finally, the mechanisms of aptamer internalization require further study. All these limitations open up prospects for future research aimed at better understanding the mechanisms of the aptamers action.

## 4. Materials and Methods

### 4.1. Cell Cultures

The human epidermoid carcinoma cell line A431, known for its high EGFR expression, was used as a positive control [31]. Passaged human glioblastoma cell cultures (G22, G23, G01, Sus/fP2 and BU73) were obtained from surgical tumour material at the N.N. Burdenko National Medical Research Centre for Neurosurgery (FSBI) (Moscow, Russia) according to a previously published methodology [48,49]. Cell cultures derived from glioblastoma tumour tissue were used with approval from the Ethics Committee of the Burdenko National Medical Research Centre for Neurosurgery. Confirmation of the purity and authenticity of cultures was carried out comprehensively at all stages of the experiment: histopathological analysis of the original tissue was carried out to confirm the presence of tumour cells and the absence of significant contamination with normal brain cells; the identity of the cell cultures was confirmed by STR profiling upon receipt and after every 10 passages. The presence of mycoplasma was tested by PCR using the MycoTrace Kit (Sigma-Aldrich, St. Louis, MI, USA) every 4 weeks. All cultures used in this study were negative for mycoplasma. Additionally, cell morphology was assessed using phase-contrast microscopy at each passage.

Primary cell cultures were maintained in DMEM/F12 growth medium (Gibco, Grand Island, NY, USA) supplemented with sodium pyruvate, 10% fetal bovine serum (FBS; Thermo Fisher Scientific, Waltham, MA, USA), 1% HEPES solution (Thermo Fisher Scientific), 1% GlutaMAX (Thermo Fisher Scientific), and 1% antibiotic–antimycotic solution (penicillin/streptomycin; Gibco, USA). Cells were incubated at 37 °C in a CO_2_ incubator (BINDER C150, Tuttlingen, Germany). For passaging, cells were rinsed with phosphate-buffered saline (PBS) and detached using 0.25% trypsin. Trypsin activity was neutralized by the addition of 1 mL of growth medium. The cell suspension was then centrifuged for 5 min at 1000 rpm, after which the supernatant was discarded, and fresh medium was added. Depending on the experimental requirements, cells were either reseeded into new flasks for continued culture and subsequent cryopreservation or plated for downstream experimental assays.

### 4.2. Aptamer Sequences

The sequences of aptamers used in this work are shown in Table 2. Aptamer synthesis was performed by Evrogen LLC (Moscow, Russia).

### 4.3. Investigation of Effect of Aptamers GR20 and U31 on Gene Expression in Human Glioblastoma Cultures G01 and Sus/fP2

Cells treated with aptamers at a final aptamer concentration of 10 µM were incubated for 72 h. Control cell cultures were incubated without aptamer addition. At the end of incubation, cells were detached from culture flasks using trypsin and washed twice with PBS buffer. Total RNA was isolated using TRI REAGENT^®^ (MRC, Cincinnati, OH, USA) according to the manufacturer’s instructions. The isolated RNA was dissolved in diethyl pyrocarbonate (DEPC)-treated water, and RNA concentration was measured using a Nano-500 spectrophotometer (ALLSHING, Nanjing, China). First-strand cDNA synthesis was performed using the MMLV RT kit (Evrogen, Russia) following the manufacturer’s protocol. Briefly, 2 µg of total RNA and 2 µL of oligo(dT)16 primer (20 µM) were used in a total reaction volume of 20 µL. Reverse transcription was carried out for 40 min at 41 °C. The resulting cDNA was subsequently used as a template for quantitative real-time PCR (qRT-PCR).

Expression levels of the genes *CD133*, *CD44*, *EGFR*, *LEF1*, *OCT4*, *PDGFRα*, and *TP53* were analyzed using a CFX96 Touch Real-Time PCR system (Bio-Rad, Hercules, CA, USA). Amplification was performed using the “Reagent Kit for Real-Time PCR in the Presence of SYBR Green I R-402” (Sintol, Moscow, Russia). The PCR protocol included an initial denaturation step at 95 °C for 5 min, followed by 40 cycles of denaturation at 95 °C for 10 s and combined annealing/extension at 60 °C for 30 s. Gene-specific primers were designed for each target gene. Relative gene expression levels were normalized using three reference genes: *GAPDH*, *GUSB*, and *HPRT*. All measurements were performed in triplicate. Primer sequences are listed in Table 3.

### 4.4. MTS Assay

GBM cells were seeded onto 96-well plates (Greiner, Pleidelsheim, Germany) at 2000 cells per well in 200 µL of DMEM/F12 growth medium (Gibco, USA) and cultured for 48 h at 37 °C in a humidified atmosphere with 5% CO_2_. Culture medium was removed from the wells and the aptamer was added at 100 µL per well at a final concentration of 10 µM. Five replicates were used for each aptamer. Prior to addition, oligonucleotides were heated for 5 min at 95 °C followed by slow cooling to room temperature. Cells treated with aptamers were incubated for 72 h at 37 °C in a humidified atmosphere with 5% CO_2_. Treatment medium were removed, cells were washed with PBS (Gibco, USA) and cultured for 24 h in 100 µL of fresh growth medium. 20 µL of MTS reagent (Promega, Madison, WI, USA) was added to 100 µL of growth medium. The plate was incubated for 2 h at 37 °C in a humidified atmosphere with 5% CO_2_. Cells that were not treated with oligonucleotides were used as controls. Untreated cells served as the control group, while wells containing only growth medium were used as blank controls. Optical density was measured using a FLUOstar Omega multimodal reader (BMG Labtech, Ortenberg, Germany) at wavelengths of 490–540 nm with Omega-Control software (version 5.70, BMG LABTECH GmbH, Ortenberg, Germany). Data were analyzed using Omega-Data Analysis software. Graphical results are presented as the percentage change in cellular metabolic activity, an indirect indicator of cell proliferation, in response to oligonucleotide treatment relative to the control group. Statistical significance of the observed differences was evaluated using the non-parametric Mann–Whitney test.

### 4.5. Flow Cytometry

To assess binding of FAM-labelled EGFR aptamers (FAM-GR20 and FAM-U31) to human glioblastoma cells, 1.5 × 10^5^ cells were seeded onto 25 cm^2^ culture flasks and incubated in DMEM/F12 growth medium with 10% FBS at 37 °C in a humidified atmosphere with 5% CO_2_. After 48 h of culture, harvested cells were washed with PBS and resuspended in 500 µL of 1× binding buffer. Cells were stained with 1 µM solutions of FITC-labelled anti-EGFR antibodies and 1 µM solutions of aptamers (FAM-GR20) and (FAM-U31) at room temperature in the dark for 30 min. The resulting samples were analyzed by flow cytometry on a CytoFLEX S (Beckman Coulter, Brea, CA, USA).

### 4.6. Aptacytochemical Analysis

Confocal microscopy was used to analyze the accumulation of FAM-labelled aptamers U31 and GR20 in human glioblastoma cells G22 and G01 at intervals of 30 min to 2 h (in vitro). Cell cultures (10,000 cells per well) were cultured in DMEM/F12 nutrient medium (Paneco, Moscow, Russia) supplemented with L-glutamine (500 mg/L) and 10% FCS (Paneco, Russia) in 4-well plates with integrated cover glasses (SPL Lifesciences, Pocheon-si, Republic of Korea) for 1–3 days at 37 °C. FAM-labelled aptamers were added to human glioblastoma cell cultures at a final concentration of 1 µM; incubation time depended on the experimental conditions (30, 60 or 120 min). Cells were washed three times with phosphate-buffered saline (PBS) (Sigma, USA) and fixed with 4% buffered paraformaldehyde (Sigma, USA) for 30 min at +4 °C. After fixation, cells were washed three times with three volumes of PBS for 5 min each. Cell nuclei were stained with bisbenzimide solution (Hoechst 33342, Sigma, dilution 1:500 in PBS). Cells were mounted in polyvinyl alcohol-based mounting medium Mowiol 4-88 (Cat. No. 9002-89-5, Sigma-Aldrich, Taufkirchen, Germany) with 1% DABCO antioxidant for 8 h at +4 °C, then for 24 h at room temperature.

### 4.7. Immunofluorescence Analysis

Human glioblastoma cell cultures G01 and Sus/fP2 were cultured in DMEM/F12 nutrient medium (Paneco, Russia) supplemented with L-glutamine (500 mg/L) and 10% FCS (Paneco, Russia) in 4-well plates (10,000 cells per well) containing cover slips (SPL Lifesciences, Pocheon-si, Republic of Korea) for 1–3 days at 37 °C. Cells were treated with aptamer U31 or GR20 at a concentration of 10 µM for 72 h. Cells were then fixed with 4% formaldehyde for 30 min. After fixation, cells were washed with three volumes of PBS and incubated in solutions of primary antibodies against KI-67 (ab15580, Abcam, Waltham, MA, USA), CD133 (Cat. No. SAB4701066, Sigma, USA; dilution 1:20), Nestin (Cat. No. Ab105389, Abcam, USA; dilution 1:50), βIII-tubulin (Cat. No. Ab18207, Abcam, USA; dilution 1:100), L1CAM (Cat. No. MA-14-140, ThermoFisher, Waltham, MA, USA; dilution 1:20), Sox2 (Cat. No. sc-17320, Santa Cruz Biotechnology, Santa Cruz, CA, USA; dilution 1:20), EGFRvIII (Cat. No. MAB1126, Thermo Scientific, Waltham, MA, USA; dilution 1:100), p53 (Cat. No. Ab1101, Abcam, USA; dilution 1:50), CD44 (Cat. No. 14-044-082, Invitrogen, Waltham, MA, USA; dilution 1:100) for 2 h, followed by three PBS washes of 5 min each. Cells were incubated with Cy2-labelled secondary antibodies at 1:100 dilution (Cat. No. 715-225-151, Jackson ImmunoResearch, Ely, UK) or Cy5-labelled secondary antibodies at 1:100 dilution for 60 min at room temperature. Cell nuclei were stained with bisbenzimide solution (Hoechst 33342, Sigma, dilution 1:500 in PBS). Cells were mounted in polyvinyl alcohol-based mounting medium Mowiol 4-88 (Cat. No. 9002-89-5, Sigma-Aldrich, Germany) with 1% DABCO antioxidant for 8 h at +4 °C, then for 24 h at room temperature. Samples were examined on a Carl Zeiss LSM-710 series laser scanning confocal microscope complex (Oberkochen, Germany) with a short-pulse femtosecond infrared tuneable laser (800–1500 nm) for multiphoton fluorescence excitation.

### 4.8. Molecular Modelling

Secondary structure models of U31 were proposed using the Vienna RNA package and the DNA parameter set [32]. Initial three-dimensional structural models of aptamers were built using 3dRNAserver [50]. Molecular dynamics simulations were performed in an explicit solvent in the presence of ions providing ionic strength and of structural significance: potassium cations. Systems were prepared using the AMBER14SB-OL15 force field [51,52] with modified Joung and Cheatham (JC) parameters for K^+^, Na^+^ and Cl^−^ ions [49]. Ion parameters were taken from a previously published study [53,54]. The numerical values reported in the article involved use of the AMBER package for molecular dynamics simulation; it was therefore necessary to convert parameter values for use in GROMACS [55]. The structure of each aptamer was optimized in vacuum using steepest descent, and the solvent environment was restored using the TIP3P water model at an ionic strength of 0.14 M. For each aptamer, 2 µs trajectories were calculated with a leap-frog integrator step of 2 fs. Electrostatic interactions were calculated using the Particle Mesh Ewald (PME) method.

### 4.9. Statistics

Statistical analyses (multiple unpaired *t*-tests) were conducted using GraphPad Prism 9.0.0 software (GraphPad Software, San Diego, CA, USA). The data are presented as the means ± SD from a minimum of three repeats. Statistical significance is presented as (* - *p* < 0.05) and (** - *p* < 0.01).

## 5. Conclusions

Thus, this study demonstrates that truncation of the U31 aptamer to GR20, while maintaining affinity to EGFR and internalization ability, results in a loss of antiproliferative activity, which correlates with the disappearance of the multi-loop conformation. These results contribute to the understanding of the structural determinants of aptamer functional activity and can be used for the rational design of the nucleic acid-based therapeutic molecules.

## Figures and Tables

**Table 1:** Results of cluster analysis of conformational diversity of aptamers U31 and GR20.

Cluster #	U31-ss-v1	U31-ss-v2	GR20-ss-v1	GR20-ss-v2
1	5359	1101	3927	1910
2	1194	1099	2094	1765
3	982	909	1712	1353
4	247	903	127	1246
5	90	481	31	365
6	79	266	22	159
7	45	248	20	145

**Table 2:** Sequences of aptamers used in study.

Aptamer	Nucleotide Sequence	Characteristics	References
U31	5′-ATC CAG AGT GAC GCA GCA TTT GTT TAA TAT GTT TTT TAA TTC CCC TTG TGG TGT GTT GTG GAC ACG GTG GCT TAG T-3′	EGFR-specific aptamer selected by SELEX	[27]
GR20	5′-ACG CAC CAT TTG TTT AAT ATG TTT TTT AAT TCC CCT TGT GGT GTG T-3′	Trimmed variant U31	[23]

**Table 3:** Primer sequences.

Genes Studied in This Work	Nucleotide Sequence of Forward Primers (5′→3′)	Nucleotide Sequence of Reverse Primers (5′→3′)
*CD133*	TGGATGCAGAACTTGACAACGT	ATACCTGCTACGACAGTCGTGGT
*CD44*	AGAAGGTGTGGGCAGAAGAA	AAATGCACCATTTCCTGAGA
*EGFR*	GTGACCGTTTGGGAGTTGATGA	GGCTGAGGGAGGCGTTCTC
*LEF1*	TGGCATCCCTCATCCAGCTATTGT	TGAGGCTTCACGTGCATTAGGTCA
*OCT4*	CGAAAGAGAAAGCGAACCAG	AACCACACTCGGACCACATC
*PDGFRα*	GGCATTCTTTGCAATACTGCTTAA	CATCTGCCGATAGCACAGTGA
*TP53*	TCAACAAGATGTTTTGCCAACTG	ATGTGCTGTGACTGCTTGTAGATG
*GUSB*	CTTCTCTGACAACCGACGCC	ACACCCAGCCGACAAAATGC
*HPRT*	TGAGGATTTGGAAAGGGTGT	GAGCACACAGAGGGCTACAA
*GAPDH*	AGATCCCTCCAAAATCAAGTGG	GGCAGAGATGATGACCCTTTT

## Data Availability

The original contributions presented in this study are included in the article. Further inquiries can be directed to the corresponding authors.

## Abbreviations

The following abbreviations are used in this manuscript:

EGFR	Epidermal Growth Factor Receptor
EGFRwt	wild-type EGFR
GBM	Glioblastoma

## References

[1] Louis D.N., Perry A., Wesseling P., Brat D.J., Cree I.A., Figarella-Branger D., Hawkins C., Ng H.K., Pfister S.M., Reifenberger G. (2021). The 2021 WHO Classification of Tumors of the Central Nervous System: A Summary. Neuro. Oncol..

[2] Brown N.F., Ottaviani D., Tazare J., Gregson J., Kitchen N., Brandner S., Fersht N., Mulholland P. (2022). Survival Outcomes and Prognostic Factors in Glioblastoma. Cancers.

[3] Grochans S., Cybulska A.M., Simińska D., Korbecki J., Kojder K., Chlubek D., Baranowska-Bosiacka I. (2022). Epidemiology of Glioblastoma Multiforme—Literature Review. Cancers.

[4] El Atat O., Naser R., Abdelkhalek M., Habib R.A., El Sibai M. (2022). Molecular targeted therapy: A new avenue in glioblastoma treatment. Oncol. Lett..

[5] Zhang A., Miao K., Sun H., Deng C.X. (2022). Tumor heterogeneity reshapes the tumor microenvironment to influence drug resistance. Int. J. Biol. Sci..

[6] Liu T., Zhu C., Chen X., Guan G., Zou C., Shen S., Wu J., Wang Y., Lin Z., Chen L. (2022). Ferroptosis, as the most enriched programmed cell death process in glioma, induces immunosuppression and immunotherapy resistance. Neuro. Oncol..

[7] Brennan C.W., Verhaak R.G., McKenna A., Campos B., Noushmehr H., Salama S.R., Zheng S., Chakravarty D., Sanborn J.Z., Berman S.H. (2013). The somatic genomic landscape of glioblastoma. Cell.

[8] Gan H.K., Cvrljevic A.N., Johns T.G. (2013). The epidermal growth factor receptor variant III (EGFRvIII): Where wild things are altered. FEBS J..

[9] An Z., Aksoy O., Zheng T., Fan Q.W., Weiss W.A. (2018). Epidermal growth factor receptor and EGFRvIII in glioblastoma: Signaling pathways and targeted therapies. Oncogene.

[10] Dhawan A., Manem V.S.K., Yeaney G., Lathia J.D., Ahluwalia M.S. (2023). EGFR pathway expression persists in recurrent glioblastoma independent of amplification status. Cancers.

[11] Wen P.Y., Kesari S. (2008). Malignant gliomas in adults. N. Engl. J. Med..

[12] Kumar Kulabhusan P., Hussain B., Yüce M. (2020). Current Perspectives on Aptamers as Diagnostic Tools and Therapeutic Agents. Pharmaceutics.

[13] Amundarain A., Pastor F., Prósper F., Agirre X. (2022). Aptamers, a New Therapeutic Opportunity for the Treatment of Multiple Myeloma. Cancers.

[14] Khan S., Hussain A., Fahimi H., Aliakbari F., Haj Bloukh S., Edis Z., Babadaei M., Izadi Z., Shiri Varnamkhasti B., Jahanshahi F. (2022). A review on the therapeutic applications of aptamers and aptamer-conjugated nanoparticles in cancer, inflammatory and viral diseases. Arab. J. Chem..

[15] Ni S., Zhuo Z., Pan Y., Yu Y., Li F., Liu J., Wang L., Wu X., Li D., Wan Y. (2021). Recent Progress in Aptamer Discoveries and Modifications for Therapeutic Applications. ACS Appl. Mater. Interfaces.

[16] Wang D.L., Song Y.L., Zhu Z., Li X.L., Zou Y., Yang H.T., Wang J.J., Yao P.S., Pan R.J., Yang C.J. (2014). Selection of DNA aptamers against epidermal growth factor receptor with high affinity and specificity. Biochem. Biophys. Res. Commun..

[17] Park N.J., Wang X., Diaz A., Goos-Root D.M., Bock C., Vaught J.D., Sun W., Strom C.M. (2013). Measurement of cetuximab and panitumumab-unbound serum EGFR extracellular domain using an assay based on slow off-rate modified aptamer (SOMAmer) reagents. PLoS ONE.

[18] Esposito C.L., Passaro D., Longobardo I., Condorelli G., Marotta P., Affuso A., de Franciscis V., Cerchia L. (2011). A neutralizing RNA aptamer against EGFR causes selective apoptotic cell death. PLoS ONE.

[19] Camorani S., Crescenzi E., Colecchia D., Carpentieri A., Amoresano A., Fedele M., Chiariello M., Cerchia L. (2015). Aptamer targeting EGFRvIII mutant hampers its constitutive autophosphorylation and affects migration, invasion and proliferation of glioblastoma cells. Oncotarget.

[20] Cheng S., Jacobson O., Zhu G., Chen Z., Liang S.H., Tian R., Yang Z., Niu G., Zhu X., Chen X. (2019). PET imaging of EGFR expression using an 18F-labeled RNA aptamer. Eur. J. Nucl. Med. Mol. Imaging.

[21] Dzarieva F., Pavlova S., Fab L., Shamadykova D., Revishchin A., Alekseeva A., Kopylov A., Pronin I., Pavlova G. (2026). New Player in the Field of Glioblastoma Therapy: EGFRvIII-Specific Gol1 Aptamer Shows a Great Therapeutic Potential. Pharmaceutics.

[22] Golovin A., Dzarieva F., Rubetskaya K., Shamadykova D., Usachev D., Pavlova G., Kopylov A. (2025). In Silico Born Designed Anti-EGFR Aptamer Gol1 Has Anti-Proliferative Potential for Patient Glioblastoma Cells. Int. J. Mol. Sci..

[23] Zavyalova E., Turashev A., Novoseltseva A., Legatova V., Antipova O., Savchenko E., Balk S., Golovin A., Pavlova G., Kopylov A. (2020). Pyrene-modified DNA aptamers with high affinity to wild-type EGFR and EGFRvIII. Nucleic Acid. Ther..

[24] Kratschmer C., Levy M. (2018). Targeted delivery of auristatin-modified toxins to pancreatic cancer using aptamers. Mol. Ther. Nucleic Acids.

[25] Huang L., Dong Y., Li J., Yang X., Li X., Wu J., Huang J., Zhang Q., Wan Z., Hu S. (2025). Epidermal Growth Factor Receptor (EGFR)-Targeting Peptides and Their Applications in Tumor Imaging Probe Construction. Biology.

[26] Hays E.M., Duan W., Shigdar S. (2017). Aptamers and glioblastoma: Their potential use for imaging and therapeutic applications. Int. J. Mol. Sci..

[27] Wu X., Liang H., Tan Y., Yuan C., Li S., Li X., Li G., Shi Y., Zhang X. (2014). Cell-SELEX aptamer for highly specific radionuclide molecular imaging of glioblastoma in vivo. PLoS ONE.

[28] Moiseenko V.L., Antipova O.M., Rybina A.A., Mukhametova L.I., Eremin S.A., Pavlova G.V., Kopylov A.M. (2024). Post-selection design of aptamers: Comparative study of affinity of DNA aptamers to the recombinant extracellular domain of human epidermal growth factor receptor. Biochemistry.

[29] Dzarieva F.M., Shamadykova D.V., Sluchanko O.V., Pavlova S.A., Fab L.V., Ryabova A.V., Panteleev D.Y., Kopylov A.M., Usachev D.Y., Golovin A.V. (2024). Specificity of Aptamers U2 and Gol1 to EGFR-Positive Human Glioblastoma Cells in Vitro. Neurosci. Behav. Physi..

[30] Kolesnikova V., Revishchin A., Fab L., Alekseeva A., Ryabova A., Pronin I., Usachev D.Y., Kopylov A., Pavlova G. (2024). GQIcombi application to subdue glioma via differentiation therapy. Front. Oncol..

[31] Zhang F., Wang S., Yin L., Yang Y., Guan Y., Wang W., Xu H., Tao N. (2015). Quantification of Epidermal Growth Factor Receptor Expression Level and Binding Kinetics on Cell Surfaces by Surface Plasmon Resonance Imaging. Anal. Chem..

[32] Turner D.H., Mathews D.H. (2010). NNDB: The nearest neighbor parameter database for predicting stability of nucleic acid secondary structure. Nucleic Acids Res..

[33] Zhang Y., Xiong Y., Xiao Y. (2022). 3dDNA: A computational method for building 3D structures of DNA. Molecules.

[34] Barducci A., Bussi G., Parrinello M. (2008). Well-tempered metadynamics: A smoothly converging and tunable free-energy method. Phys. Rev. Lett..

[35] Bottaro S., Bussi G., Pinamonti G., Reißer S., Boomsma W., Lindorff-Larsen K. (2019). Barnaba: Software for analysis of nucleic acid structures and trajectories. RNA.

[36] Yu X., Orr C.M., Chan H.T.C., James S., Penfold C.A., Kim J., Inzhelevskaya T., Mockridge C.I., Cox K.L., Essex J.W. (2023). Reducing affinity as a strategy to boost immunomodulatory antibody agonism. Nature.

[37] Cowles S.C., Sheen A., Santollani L., Lutz E.A., Lax B.M., Palmeri J.R., Freeman G.J., Wittrup K.D. (2022). An affinity threshold for maximum efficacy in anti-PD-1 immunotherapy. mAbs.

[38] Song L., Wang Y., Guo Y., Bulale S., Zhou M., Yu F., He L. (2024). Engineering aptamers to enhance their interaction with protein target for selective inhibition of cell surface receptors. Int. J. Biol. Macromol..

[39] Sacks D., Baxter B., Campbell B.C.V., Carpenter J.S., Cognard C., Dippel D., Eesa M., Fischer U., Hausegger K., Hirsch J.A. (2018). Multisociety Consensus Quality Improvement Revised Consensus Statement for Endovascular Therapy of Acute Ischemic Stroke. Int. J. Stroke.

[40] Wirmer-Bartoschek J., Ferner J.P., Heckel A., Schwalbe H. (2026). Dissecting the Chemical and Thermal Stabilities of Tetrads in G-Quadruplexes to Derive a Structure-Activity Relation for a Thrombin-Binding DNA G-Quadruplex Aptamer. Chembiochem.

[41] Song X., Yu H., Sullenger C., Gray B.P., Yan A., Kelly L., Sullenger B. (2023). An Aptamer That Rapidly Internalizes into Cancer Cells Utilizes the Transferrin Receptor Pathway. Cancers.

[42] Antipova O., Moiseenko V., Dzarieva F., Savchenko E., Pronin I., Pavlova G., Kopylov A. (2024). Corrigendum to “Varieties of Interactions of anti-CD133 Aptamers with Cell Cultures from Patient Glioblastoma”. SLAS Discov..

[43] Moiseenko V.L., Antipova O.M., Pavlova S.A., Pronin I.N., Pavlova G.V., Kopylov A.M. (2024). Is it possible to detect surface antigen CD133 on patient-derived glioblastoma continuous cell cultures using fluorescent aptamers?. Burdenko’s J. Neurosurg..

[44] Saito N., Hirai N., Aoki K., Suzuki R., Fujita S., Nakayama H., Hayashi M., Ito K., Sakurai T., Iwabuchi S. (2019). The Oncogene Addiction Switch from NOTCH to PI3K Requires Simultaneous Targeting of NOTCH and PI3K Pathway Inhibition in Glioblastoma. Cancers.

[45] Hadiloo K., Mostanadi P., Asadzadeh A., Taremi S., Esmaeilzadeh A. (2025). Targeting cancer stem cells with CAR-based immunotherapy: Biology, evidence, and future directions. Cancer Cell Int..

[46] Pavlova S., Fab L., Dzarieva F., Ryabova A., Revishchin A., Panteleev D., Antipova O., Usachev D., Kopylov A., Pavlova G. (2024). Unite and Conquer: Association of Two G-Quadruplex Aptamers Provides Antiproliferative and Antimigration Activity for Cells from High-Grade Glioma Patients. Pharmaceuticals.

[47] Dzarieva F.M., Pavlova S.A., Pavlova G.V., Fab L.V. (2025). Aptamers and endocytosis: The path into tumor cells. Biochem. Biophys. Res. Commun..

[48] Sliman Y.A., Samoylenkova N.S., Antipova O.M., Brylev V.A., Veryutin D.A., Sapozhnikova K.A., Alekseeva A.I., Pronin I.N., Kopylov A.M., Pavlova G.V. (2024). Covalently conjugated DNA aptamer with doxorubicin as in vitro model for effective targeted drug delivery to human glioblastoma tumor cells. Burdenko’s J. Neurosurg..

[49] Sliman Y.A., Pavlova S.A., Savchenko E.A., Samoilenkova N.S., Ovechkina A.V., Demyanovich A.V., Antipina N.A., Golanov A.V., Usachev D.Y., Pavlova G.V. (2025). Comparison of the effect of DNA aptamers U31, GR20, GR200 and their combination with radiation therapy on the viability of human glioblastoma cells G22 and Sus\fP2. Burdenko’s J. Neurosurg..

[50] Wang J., Wang J., Huang Y., Xiao Y. (2019). 3dRNA v2.0: An Updated Web Server for RNA 3D Structure Prediction. Int. J. Mol. Sci..

[51] Dietschreit J.C., Diestler D.J., Knapp E.W. (2016). Chemically realistic tetrahedral lattice models for polymer chains. J. Chem. Theory Comput..

[52] Lindorff-Larsen K., Piana S., Palmo K., Maragakis P., Klepeis J.L., Dror R.O., Shaw D.E. (2010). Improved side-chain torsion potentials for the Amber ff99SB protein force field. Proteins.

[53] Joung I.S., Cheatham T.E. (2008). Determination of alkali and halide monovalent ion parameters for use in explicitly solvated biomolecular simulations. J. Phys. Chem. B.

[54] Carvalho F.S., McMahon A., Case D.A., Luchko T. (2026). Development of an Optimized Parameter Set for Monovalent Ions in the Reference Interaction Site Model of Solvation. J. Chem. Phys..

[55] Abraham M., Murtola T., Schulz R., Páll S., Smith J., Hess B., Lindahl E. (2015). GROMACS: High performance molecular simulations through multi-level parallelism from laptops to supercomputers. SoftwareX.

